# Predicting Patients Requiring Treatment for Depression in the Postpartum Period Using Common Electronic Medical Record Data Available Antepartum

**DOI:** 10.1016/j.focus.2023.100100

**Published:** 2023-04-27

**Authors:** Colin Wakefield, Martin G. Frasch

**Affiliations:** 1 Drexel University College of Medicine, Philadelphia, Pennsylvania; 2 Department of Obstetrics & Gynecology, University of Washington, Seattle, Washington; 3 Center on Human Development and Disability, University of Washington, Seattle, Washington; 4 Health Stream Analytics, Seattle, Washington

**Keywords:** Artificial intelligence, perinatal depression, postpartum depression, health outcomes, preventive medicine

## Abstract

**Introduction:**

Depression requiring treatment in the postpartum period significantly impacts maternal and neonatal health. Although preventive management of depression in pregnancy has been shown to decrease the negative impacts, current methods for identifying at-risk patients are insufficient. Given the complexity of the diagnosis and interplay of clinical/demographic factors, we tested whether machine learning techniques can accurately identify at-risk patients in the postpartum period.

**Methods:**

This is a retrospective cohort study of the NIH Nulliparous Pregnancy Outcomes Study: Monitoring Mothers-to-Be, which enrolled 10,038 nulliparous people. The primary outcome was depression in the postpartum period. We constructed and optimized 4 machine learning models using distributed random forest modeling and 1 logistic regression model on the basis of the NIH Nulliparous Pregnancy Outcomes Study: Monitoring Mothers-to-Be dataset. Model 1 utilized only readily obtainable sociodemographic data. Model 2 added maternal prepregnancy mental health data. Model 3 utilized recursive feature elimination to construct a parsimonious model. Model 4 further titrated the input data to simplify prepregnancy mental health variables. The logistic regression model used the same input data as Model 3 as a proof of concept.

**Results:**

Of 8,454 births, 338 (4%) were complicated by depression in the postpartum period. Model 3 was the highest performing, showing the area under the receiver operating characteristics curve of 0.91 (±0.02). Models 1–3 identified the 9 variables most predictive of depression hierarchically, ranging from depression history (highest), history of mental health condition, recent psychiatric medication use, BMI, income, age, anxiety history, education, and preparedness for pregnancy (lowest). In Model 4, the area under the receiver operating characteristics curve remained at 0.79 (±0.05).

**Conclusions:**

Postpartum depression can be predicted with high accuracy for individual patients using antepartum information commonly found in electronic medical records. In addition, baseline mental health status and sociodemographic factors have a larger role in the postpartum period than previously understood.

## Highlights

• Postpartum depression can be predicted using machine learning.
• Predictors are from the prepartum and early antepartum periods and were easily obtainable.
• Sociodemographic characteristics are predictive of postpartum depression.
• Strong predictors are modifiable through social policy and counseling.
• We share the open source R code to facilitate further study of the Nulliparous Pregnancy Outcomes Study: Monitoring Mothers-to-Be dataset.

## INTRODUCTION

In the U.S., as many as 1 in 7 pregnant people will develop depression in the postpartum period.^1^ Yet, only 50% of affected women are diagnosed, and even fewer receive appropriate treatment.^2^ Postpartum depression is often underdiagnosed and undertreated, owing to stigma and mislabeling as the mommy blues, in a failure to recognize that untreated postpartum depression (PPD) significantly impacts both mother and child.

PPD impacts patients differently depending on the time of onset. Prepartum onset increases the risk of preterm birth, pre-eclampsia, low birth weight, maternal suicide, and poor cognitive and behavioral trajectories in the fetus.^3^ The maternal suicide risk associated with prepartum onset should not be underestimated because it surpasses both hemorrhage and hypertensive disorders as the leading cause of maternal mortality during pregnancy.^4^ PPD carries the additional risk of negatively impacting child safety owing to depressive symptoms in the mother. Postpartum presentation has been linked to less breastfeeding, poor adherence to infant safety recommendations, fewer well-child visits, and an overall increased risk of child neglect. Mothers during this period are less likely to take care of their own needs and have a pronounced risk of self-harm because suicide accounts for 20% of deaths in moms within the year after birth.^1^

The long-term consequences of PPD are often underestimated. Feelings of intense sadness, anxiety, and anhedonia impede bonding with the infant, resulting in cognitive, behavioral, and emotional developmental delays for the child, as well as delays in social and communication skills.^1^ Such delays can have lifelong ramifications for the child and pose a real threat to their capacity for social adjustment.

Fortunately, early detection and subsequent preventive interventions are highly efficacious in mitigating the consequences of PPD. In 2019, the U.S. Preventive Services Task Force (USPSTF) conducted a meta-analysis, finding that interpersonal therapy and/or cognitive behavioral therapy (CBT) in at-risk pregnant people is associated with a 39% reduction in the likelihood of developing PPD. In specific at-risk populations, the associated risk reductions are as high as 53%.^5^

Even though the impacts of PPD can be profound and early intervention has been shown to significantly reduce the incidence, there are no tools available for easy identification of at-risk patients. The existing early intervention paradigm is based on a USPSTF Grade B recommendation wherein counseling is provided to women with 1 or more established risk factors for PPD, such as a history of depression or recent intimate partner violence.^6^ The current approach for identifying at-risk patients is crude at best and represents an opportunity to mobilize available technologies such as machine learning (ML) in a way that can improve health outcomes. The lack of effective screening tools is a public health issue because early interventions in at-risk people can minimize the morbidity and mortality of PPD. The development of a tool to stratify PPD risk and assist with resource allocation to in-need populations has the potential to greatly improve perinatal healthcare delivery.

As such, using ML models, previous groups have reported predictive models for identifying patients at risk of PPD with modest success. Over the past 14 years, there have been 18 publications presenting ML models for predicting PPD.^6^^,^^7^ A common issue among these models has been the failure to accurately predict PPD using only readily obtainable data. Previous models have attempted to incorporate input data that are neither uniform in nature nor easily collected, such as psychological resilience, dental hygiene, and 5-HTT-GC levels.^8^, ^9^, ^10^ Previous models have routinely incorporated late gestation or postpartum data, greatly reducing their predictive value. A substantial number of PPD cases arise early in pregnancy (first trimester: 7%, second trimester: 13%), thus models relying on late gestation/postpartum data fail to capture substantial patient populations.^11^ Moreover, for PPD prediction models to be effective, at-risk patients must be identified early enough in pregnancy for CBT to take effect. Therefore, a PPD prediction model needs to use input data that can be easily gathered early in or before pregnancy, something that only 1 previous model has found moderate success in doing.^6^

Given the morbidity and mortality of PPD, there is a clear need for effective screening tools to quantify individual patient risk for PPD. The complexity of the diagnosis and interplay of clinical/demographic factors makes it difficult for clinicians to individually identify at-risk patients. Therefore, our team tested whether ML techniques can accurately identify patients at risk of PPD using only prepregnancy data found in electronic medical records (EMRs).

## METHODS

### Study Sample

To test whether ML techniques can accurately identify patients at risk of PPD requiring treatment, our team performed a retrospective cohort analysis of the Nulliparous Pregnancy Outcomes Study: Monitoring Mothers-to-Be (nuMoM2b) data set. The nuMoM2b study is a prospective cohort study administered by the NIH, in which 10,038 nulliparous women with singleton pregnancies were enrolled from geographically diverse hospitals affiliated with 8 clinical centers. It is unique because it was designed to investigate the interrelated mechanisms of common adverse pregnancy-related outcomes. Through its design, the nuMoM2b study subsequently created one of the largest public data sets from which the relationship between hundreds of sociodemographic variables and different pregnancy-related outcomes can be investigated. The data can be obtained through the NICHD DASH (Eunice Kennedy Shriver National Institute of Child Health and Human Development Data and Specimen Hub) portal.^12^

Full details of the study protocol have been previously published.^13^^,^^14^ In brief, women were eligible for enrollment if they had a viable singleton gestation, had no previous pregnancy that lasted more than 20 weeks of gestation, and were between 6 weeks 0 days gestation and 13 weeks 6 days gestation at recruitment. Exclusion criteria were maternal age <13 years, history of 3 or more spontaneous abortions, current pregnancy complicated by a suspected fatal fetal malformation, known fetal aneuploidy, assisted reproduction with a donor oocyte, multifetal reduction, or plan to terminate the pregnancy. Patients were also excluded if they were already participating in an intervention study anticipated to influence pertinent maternal or fetal outcomes, were previously enrolled in the nuMoM2b study, or were unable to provide informed consent. A common protocol and manual of operations were used for all aspects of the study. Each site's local governing IRB approved the study, and all women provided informed written consent before participation.

### Measures

Participant data were collected by trained research personnel during 3 antepartum study visits. These visits were scheduled to occur between 6 weeks 0 days and 13 weeks 6 days gestation (Visit 1), 16 weeks 0 days and 21 weeks 6 days gestation (Visit 2), and 22 weeks 0 days and 29 weeks 6 days gestation (Visit 3). A woman's self-identified race and ethnicity were categorized as non-Hispanic White, non-Hispanic Black, Hispanic, Asian, or other. At least 30 days after delivery, trained and certified chart abstractors reviewed the medical records of all participants and recorded final maternal and birth outcomes. Owing to inconsistencies in Edinburgh Postnatal Depression Scale documentation across the nuMoM2b study, our team used treatment of depression in the postpartum period as a proxy for the diagnosis of PPD (variable CMAE04a1c in the nuMoM2b data set). In comparison with the Edinburgh Postnatal Depression Scale scoring, chart abstractors recorded the status of each patient in regard to being treated for depression in the postpartum period, which provided a uniform PPD outcome for use in our ML models. The list of predictor variables used in the present ML models is provided in Table 1. For instances of missing data, no imputation was undertaken; rather, cases with missing data for the outcome variable were excluded.

**Table 1:** Description of predictor variables

Predictor variable	Description
Race	Self-identified race: American Indian/Alaska Native, Asian, Native Hawaiian/other Pacific Islander, Black/African American, White, more than 1 race
Hispanic	Hispanic ethnicity as defined by NIH enrollment
BMI	Numeric BMI (kg/m^2^) value
BMI Cat	BMI (kg/m^2^) range: ≤18.5, 18.5 to <25, 25 to <30, 30 to <35, ≥35
Education	Education status ranging from 8th grade or less to doctoral
GravCat	Number of previous pregnancies: 1, 2, ≥3
Smoke Cat1	Has patient ever smoked tobacco: Yes, No
Smoke Cat2	Has patient smoked tobacco in 3 months leading up to pregnancy: Yes, No
Smoke Cat3	Number of cigarettes per day if patient did smoke in 3 months leading up to pregnancy: <20 per day, 20−40 per day, >40 per day
Ins_Govt	Health care paid for by government insurance
Ins_Military	Health care paid for by military insurance
Ins_Comm	Health care paid for by commercial health insurance
Ins_Pers	Health care paid for by the patient
Ins_Othr	Health care paid for by other
PctFedPoverty	Patient income as a percentage relative to the Federal Poverty Level
Poverty	Patient income as a percentage of Federal Poverty Level by category: <100%, 100%−200%, >200%
Age at V1	Age at visit in numerical years
V1AD02g	Before you got pregnant, did a doctor, nurse, or other health care worker talk to you about the following regarding how to prepare for a healthy pregnancy and baby? - Getting counseling or treatment for depression or anxiety
CMAE04a1a	Does the patient have a history of depression prior to pregnancy: Yes, No
CMAE04a2a	Does the patient have a history of anxiety prior to pregnancy: Yes, No
VXXB01bb_V1a	Does the patient have an existing mental health condition: Yes, No
VXXB01bb_V1b	At the initial visit, has the patient recently used medications for a mental health condition: Yes, No

*Note:* The predictive factors used in ML Models 2 and 3 are shown. Predictive factors used in ML Models 2 and 3 can be searched in the NIH's nuMoM2b study using the variable identifiers mentioned earlier. We have listed the corresponding description of each variable for the ease of the reader.

ML, machine learning; nuMoM2b, Nulliparous Pregnancy Outcomes Study: Monitoring Mothers-to-Be.

### Statistical Analysis

The analyses were performed in R 4.1.1 with h2o 3.34.0.3 (computationally efficient ML modeling framework). The training was done on 80% of the data, tenfold cross-validation was performed on 20% of the held-out data, and the model performance is reported from that unseen validation dataset. The code, results, and notebook^15^ for validation and extension of the presented solution have been deposited as open source.^16^ The following results are reported for the distributed random forest (DRF) ML models and logistic regression models. ML model performances were compared using a 2-sided *t*-test for related samples, and results were corrected for multiple comparisons using the Holm procedure.

## RESULTS

An ML titration approach was deployed to (1) optimize performance and (2) maximize model practicality by minimizing the number of variables included. Of the 10,038 women enrolled in this prospective cohort, 9,470 were eligible for the present analysis after exclusion for lack of pregnancy outcome data, lack of race−ethnicity data, and pregnancy terminations/fetal death. Among eligible women, 5,721 (60.4%) were non-Hispanic White, 1,307 (13.8%) were non-Hispanic Black, 1,586 (16.7%) were Hispanic, 379 (4.0%) were Asian, and 477 (5.0%) were of another race or ethnicity. After further exclusion due to a lack of follow-up visits, 8,545 participants’ data were available for the ML training to predict PPD, which was reported in 338 cases.

Titration began with Model 1, taking a simple approach that only included readily obtainable sociodemographic data (insurance, income, education, race, BMI, and smoking status) and 2 prepregnancy mental health status variables (previous post-traumatic stress disorder and previous discussion of mental health treatment with a provider). Model 1 performed with an area under the curve (AUC) of 0.63 (±0.07) (Table 2). Interestingly, adding prepartum hypertension and diabetes mellitus information did not improve model performance. In Model 2, another extreme was tested: use all relevant prepregnancy mental health variables available in the nuMoM2b data set (cf. the notebook^15^). The inclusion of 111 variables in Model 2 considerably improved the predictive accuracy because it achieved an AUC of 0.93 (±0.02) (Table 2). Model 3 was derived by pruning Model 2 to only include the 9 most contributing variables: history of depression before pregnancy (yes/no), history of mental health condition before pregnancy (yes/no), recent medications for a mental health condition at the initial visit (yes/no), BMI, income, age, history of anxiety before pregnancy (yes/no), education, and discussing with a provider about preparedness for pregnancy before becoming pregnant (yes/no). Pruning to yield Model 3 resulted in an overall best performing model with an AUC of 0.91(±0.02) (Table 2). Model 4 further titrated Model 3 to simplify the input variables to 6 variables in the following descending order of importance: history of mental health condition before pregnancy (yes/no), BMI, recent medications for a mental health condition at the initial visit (yes/no), age, income, and education. Model 4 yielded an AUC of 0.79 (±0.05) (Table 2).

**Table 2:** ML Model Overview: DRF Models and Logistic Regression

Model	Variables	AUC (±SD)
Model 1	Six sociodemographic variables (insurance, income, education, race, BMI, and smoking status) and 2 prepregnancy mental health variables (previous PTSD and previous discussion on mental health treatment)	0.63 (±0.07)
Model 2	All relevant prepregnancy data in nuMoM2b study (111)	0.93 (±0.02)
Model 3	Nine highest contributing variables	0.91 (±0.02)
Model 4	Removing depression- and anxiety-specific variables from Model 3	0.79 (±0.05)
Logistic regression	Nine highest contributing variables	0.93 (±0.03)

*Note:* An overview of Models 1–4 and logistic regression is shown. DRF ML Models 1–4 predict PPD. The variables used in Models 1–4 were identified using the titration approach outlined in the methods section. A logistic regression was conducted on the 9 variables identified by DRF Model 2 and used in DRF Model 3 (cf. Figure 2). No statistical difference was identified between the performance of Models 2 and 3 (*p*=0.11) or Model 2 and the logistic regression (*p*=0.90). All other head-to-head model comparisons showed statistically significant differences, achieving *p*<0.02.

AUC, area under the curve; DRF, distributed random forest; ML, machine learning; nuMoM2b, Nulliparous Pregnancy Outcomes Study: Monitoring Mothers-to-Be; PTSD, post-traumatic stress disorder.

**Figure 1:**
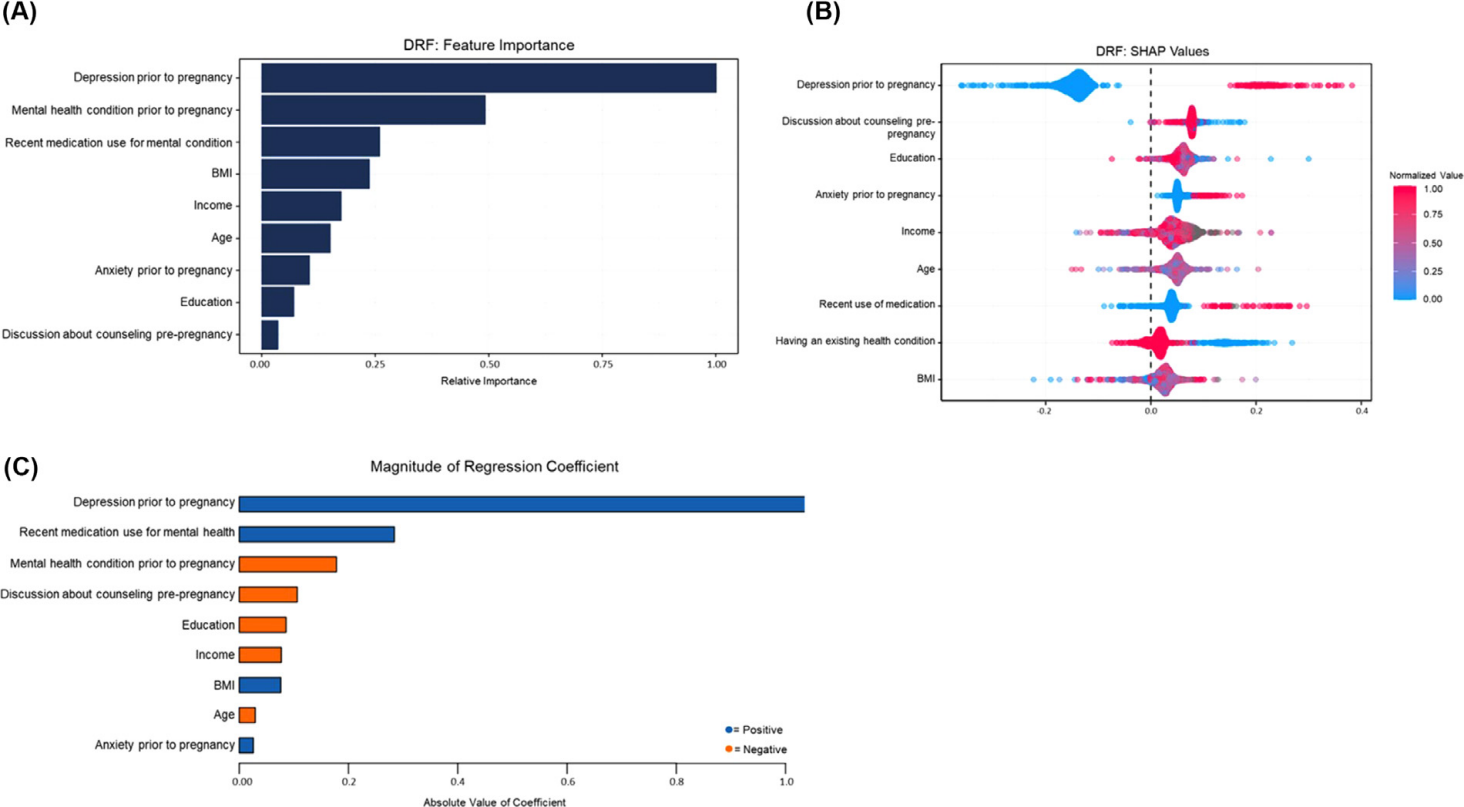
Contribution of the individual variables in the prediction. **(A)** The 9 most contributing factors to the development of PPD requiring treatment, listed in descending order of relative importance: history of depression before pregnancy, history of mental health condition before pregnancy, recent medications for a mental health condition at the initial visit, BMI, income, age, history of anxiety before pregnancy, education, and discussing with a provider about preparedness for pregnancy before becoming pregnant. **(B)** SHAP analysis reflecting the relative importance of the 9 most contributing variables in the DRF (Models 1–4) PPD models on the individual level. **(C)** Regression coefficient values for the logistic regression model. Notably, existing mental health conditions, discussions about counseling before pregnancy, high education, higher income, and older age are all negatively associated with PPD development.

DRF, distributed random forest; PPD, postpartum depression; SHAP, SHapley Additive exPlanations.

To further elucidate the directions of the contributions of the predictive features, a logistic regression analysis was conducted using the same 9 variables identified by the DRF Model 3 as most contributing (Figure 1). The logistic regression yielded an AUC of 0.93(±0.03) (Table 2 and Figure 1). No significant difference was found between the performance of Model 3 (DRF) and the logistic regression model.

## DISCUSSION

PPD significantly impacts maternal and neonatal health as well as the family unit as a whole. Affected mothers are at an increased risk of child neglect, marital conflict, mood disorder development, and poor attachment to the infant. Children born to mothers with PPD show delays in cognitive, psychosocial, motor skills, and social skills development. More importantly, PPD is a significant risk factor for infanticide and suicide, with suicide being the leading cause of maternal death in the initial postpartum year.^17^ Given the risk of PPD and the efficacy of existing preventative treatment modalities, the USPSTF has recommended focusing on the prenatal prevention of PPD rather than on postpartum screening.^6^ Yet, no individualized approaches currently exist to identify mothers at risk for PPD.

We present 4 DRF ML models and a logistic regression ML model that are capable of identifying patients at high risk for developing PPD requiring treatment, using readily available prepregnancy information found in the EMRs.

Of the ML models constructed by our group, Model 3 was the best performing, correctly identifying PPD requiring treatment in 91% of patients while only using 9 factors related to medical history and sociodemographics (Table 1 and Figure 1). Moreover, all 9 factors (history of depression before pregnancy, history of mental health condition before pregnancy, recent medications for a mental health condition at the initial visit, BMI, income, age, history of anxiety before pregnancy, education, and discussing with a provider about preparedness for pregnancy before becoming pregnant) are easily obtained before pregnancy, an important consideration when developing a clinical tool intended to provide clinicians adequate time for detection and intervention (Figure 1).

It is estimated that 43.7% of PPD cases arise during pregnancy; thus, in conjunction with the shift in focus toward prenatal prevention of PPD, our findings make an important contribution to the existing body of literature because there is currently only 1 published model that successfully predicts PPD using exclusively antepartum data.^6^ The Munk-Olsen group used an approach similar to ours and developed a PPD prediction model from prepartum data that achieved an AUC of 0.804.^6^ Most previously developed predictive models for PPD have failed to use exclusively prepregnancy or even early pregnancy data, limiting their therapeutic potential. This is because preventive intervention for PPD is largely through CBT, an evidence-based, longitudinally time-intensive exercise intended to modify patients’ approach to processing stress.^18^ Successful implementation of CBT can decrease PPD incidence by up to 53%.^5^ However, patients identified as being at risk for developing PPD and requiring treatment need adequate time to participate in CBT and develop healthy coping strategies before disease onset. Therefore, to maximize the therapeutic potential of a predictive model for PPD, prepregnancy data should be used.

Our models address this need using exclusively prepregnancy data to develop a highly predictive PPD model. The sociodemographic and mental health history data points used by our ML algorithms are binary/categorical in nature and routinely found in EMRs. This approach allows for simpler integration into the modern healthcare model by limiting the need for additional data collection during patient visits. The ML code can be integrated into EMR systems, automatically pulling information gathered from intake forms and previous visits. Thus, clinicians can be provided with individual patient risk stratifications by manipulating data currently contained in the EMR.

A goal of this work was to further delineate the role of various factors in the development of PDD requiring treatment. To achieve this, Model 4 was constructed by excluding maternal prepregnancy depression and anxiety history from Model 3. Instead, general mental health characteristics (history of mental health condition before pregnancy [yes/no], recent medications for a mental health condition at the initial visit [yes/no]) were included alongside sociodemographic characteristics. The approach provided clarity on the role of prepregnancy mental health history in the development of PPD requiring treatment. Interestingly, Model 4 performed relatively well, predicting 79% of cases while only using 6 predictors (history of mental health condition before pregnancy, BMI, recent medications for a mental health condition at the initial visit, age, income, and education) (Table 1). The mild decrease in predictive accuracy from Models 3 and 4 suggests that a patient's mental health background as a whole, in addition to sociodemographic factors, plays a larger role in PPD development relative to anxiety and depression than previously understood.

The role of baseline mental health status becomes more apparent when looking at the top predictive factors for PPD identified by our models. As expected, our models identified that depression before pregnancy is the largest contributor to the development of PPD requiring treatment, substantiating the well-known role of previous depression in PPD. The second most predictive factor in the development of PPD is the presence of an existing mental health condition, whereas the third most predictive factor is the use of psychiatric medications before pregnancy (Figure 1). Interestingly, the data indicate that these latter 2 factors have inverse relationships in regard to the development of PPD. The presence of a pre-existing mental health condition negatively correlates with PPD development, likely reflecting increased access to mental resources and providers. However, the use of psychiatric medications before pregnancy is positively correlated with PPD development, corroborating the role of clinically apparent mental health conditions, namely depression, in the development of PPD. It is unclear from our data where the threshold between these 2 factors lies, but it does appear that one exists. From a public health perspective, our data support the notion that greater incorporation of preventative mental health measures into the American healthcare delivery system has the potential in and of itself to decrease the morbidity and mortality of PPD.

The role of sociodemographic factors in PPD has been documented; however, their individual relative importance has not been thoroughly established. In our algorithms, predictive accuracy is largely maintained across Models 3 and 4, highlighting the relative importance of sociodemographic factors such as BMI, income, age, and education in the development of PPD requiring treatment. All 4 models identified BMI as a highly contributing factor in the development of PPD requiring treatment, likely reflecting the physical, cognitive, and social impacts of body weight on human health. In addition, our models were able to provide evidence of the role of education and income in PPD incidence. Figure 1 shows that as both income and education levels increase, the incidence of PPD decreases. The discovery of the directional role of income and education in PPD development is not surprising but is important baseline evidence to establish to help facilitate data-driven decision making. The fact that sociodemographic factors play such a pivotal role in the pathogenesis of PPD requiring treatment is key to the formulation of public health measures to target PPD. This work provides a clear direction for policymakers to decrease the incidence of PPD. Our findings highlight that 3 of the 8 most contributing factors to PPD development (BMI, income, and education) are all modifiable through socioeconomic interventions. Policies aimed at improving income and education equity as well as access to affordable and healthy diets have the potential to decrease the morbidity and mortality associated with PPD.

This study also provides clear actionables for clinicians to mitigate the impacts of PPD. Population-based studies have linked BMI to PPD; however, the relative importance of BMI in PPD pathogenesis compared with those of other contributing factors is not well documented.^19^ Our data suggest that clinical interventions to optimize BMI are some of the most important steps to decrease patients’ risk of developing PPD requiring treatment. Lifestyle modifications as well as assisting patients in navigating social programs related to job training, education access, and Supplemental Nutrition Assistance Programs are all potential actions for clinicians to guide patients and help decrease PPD incidence.

### Limitations

This project identified prepregnancy anxiety and depression as major contributors to PPD development. However, our findings do not delineate the impact of poorly controlled versus well-controlled anxiety and depression on PPD development. Previous correlational studies have suggested a linear relationship between the severity of prepregnancy anxiety and PPD risk. Nonpregnant women with higher levels of worry as defined by the Penn State Worry Questionnaire, reflecting poorly controlled anxiety, developed more PPD symptoms.^20^ Our data provide perspective to these findings by showing the relative importance of prepregnancy anxiety and depression in PPD development in relation to nonpsychological factors such as BMI, age, education, and income. Given the correlational relationship between control of prepregnancy psychiatric illness and PPD, our predictive models could potentially also be used to identify expectant patients who may benefit from medications such as selective serotonin reuptake inhibitors to better control prepregnancy anxiety and depression in an effort to decrease PPD incidence.

Another potential limitation of this study was the use of depression requiring treatment in the postpartum period as the PPD outcome for the ML algorithms. Although the impact is not known, it should be noted that defining PPD in this manner has the potential consequence of our models missing some cases of PPD. Conversely, limiting the sensitivity of the models to only PPD cases requiring intervention has the potential to improve the clinical applicability of the algorithms. A sensitivity-limited approach prevents the identification of PPD cases that may be below the therapeutic threshold, potentially mitigating unnecessary strain on an already resource-limited mental healthcare system.

## CONCLUSIONS

PPD can be predicted with high accuracy for individual patients using antepartum information commonly found in the EMR. ML techniques offer the potential to decrease PPD incidence through early detection and subsequent CBT intervention, which has been shown to decrease PPD in at-risk populations by up to 53%. As expected, a prepregnancy history of depression is the most contributing factor to PPD development. Our models identified BMI as the most contributing sociodemographic factor in the development of PPD. Furthermore, 3 of the 8 most contributing factors identified by our predictive models (BMI, income, and education) are modifiable through social policy and clinical interventions, suggesting scalable routes to decreasing the morbidity and mortality associated with PPD.

## CRediT authorship contribution statement

**Colin Wakefield:** Conceptualization, Validation, Formal analysis, Visualization, Project administration, Writing – original draft, Writing – review & editing. **Martin G. Frasch:** Conceptualization, Methodology, Software, Validation, Formal analysis, Data curation, Writing – original draft, Writing – review & editing, Visualization, Supervision.
